# BRAF Inhibitors for BRAF V600E Mutant Colorectal Cancers: Literature Survey and Case Report

**DOI:** 10.1155/2018/8782328

**Published:** 2018-04-15

**Authors:** Yasar Subutay Peker, Mehmet Fatih Can, Ismail Hakki Ozerhan, Gokhan Yagci, Nazif Zeybek, Kutan Kavakli, Sedat Gurkok, Alper Gozubuyuk, Onur Genc, Gokhan Erdem, Ahmet Ozet, Mustafa Gerek, Yusuf Peker

**Affiliations:** ^1^Department of General Surgery, Gulhane Training and Research Hospital, University of Medical Sciences, Ankara, Turkey; ^2^Department of General Surgery, Guven Hospital, Ankara, Turkey; ^3^Department of Thoracic Surgery, Gulhane Training and Research Hospital, University of Medical Sciences, Ankara, Turkey; ^4^Department of Medical Oncology, Liv Hospital, Istanbul, Turkey; ^5^Department of Medical Oncology, Gazi University Hospital, Ankara, Turkey; ^6^Department of Otorhinolaryngology, University of Medical Sciences, Ankara, Turkey; ^7^Department of General Surgery, Gulhane Military Medical Academy, Ankara, Turkey

## Abstract

The main method of fighting against colon cancer is targeted treatment. BRAF inhibitors, which are accepted as standard treatment for V600E mutant malign melanomas, are the newest approach for targeted treatment of V600E mutant colorectal cancers. In this case report, we share our experience about the use of BRAF inhibitor vemurafenib on a V600E mutant metastatic right colon adenocarcinoma patient. A 59-year-old male with only lung multiple metastatic V600E mutant right colon cancer presented to our clinic. The patient was evaluated and FOLFOX + bevacizumab treatment was initiated, which was then continued with vemurafenib. A remarkable response was achieved with vemurafenib treatment in which the drug resistance occurred approximately in the sixth month. Even though the patient benefited majorly from vemurafenib, he died on the 20th month of the diagnosis. The expected overall survival for metastatic V600E mutant colon adenocarcinoma patients is 4.7 months. BRAF inhibitors provide new treatment alternatives for V600E mutant colorectal cancers, with prolonged overall survival. BRAF inhibitors in combination with MEK inhibitors are reported as feasible treatment to overcome BRAF inhibitor drug resistance on which phase studies are still in progress. To conclude, BRAF inhibitors alone or in combination with other drugs provide a chance for curing BRAF V600E mutant colorectal cancer patients.

## 1. Introduction

Colon cancer is a major cause of oncologic deaths all around the world, with an incidence that is expected to increase until 2035 [1]. The global medical industry, especially the pharmaceutical industry that specializes in oncology, allocates a substantial budget for this subject. The main method of fighting against colon cancer on which the industry has been focused is targeted treatment, the rising star of the last two decades. Before the clinical usage of monoclonal antibodies, which are the core of targeted therapies, coping with the side effects of conventional chemotherapies for cancer treatment was the hardest part of treatment. After the development of targeted therapy, concurrent with surgical innovation as mesocolic excision, it become a part of clinical practice which marked improvement in cure and overall survival rates of colon cancer patients.

The commonly used targeted therapies for colon cancer include angiogenesis inhibitors and growth factor inhibitors. Angiogenesis inhibitors are nonspecific for the tumor type due to the increased angiogenesis that is common in all types of tumors, but growth factor inhibitors, such as panitumumab and cetuximab customized to EGFR, have a narrow range for clinical use [2]. The wild-type *K*-*RAS/N-RAS* gene is an indispensable provision for this pair to be medicated [3]. The *K-RAS* gene, representing the *RAS* gene family, is vital for the protein synthesis necessary for growth factors [4, 5]. Once *K-RAS* has mutated, in other words, the production of growth factors becomes out of control, growth factor inhibitors are no longer applicable. Nevertheless, targeted therapies give an increased chance of cure for colon cancer patients with the wild-type *RAS* gene.

Apart from the aforementioned optimistic situation, some subtypes of colon cancers, such as BRAF mutations, come with a very short overall survival rate of 4.7 months [6]. Fortunately, the incidence is 5–10% of all metastatic colorectal cancers (mCRCs), which is approximately 1% of all colorectal cancers (CRCs) [7–10]. The distribution of the BRAF V600E mutation in the Turkish population with mCRCs is reported to be 2%, which is <1% for all CRCs [11]. Despite its low incidence and short overall survival, researchers have discovered targeted therapies for BRAF-mutant CRCs.

The BRAF gene plays an important role in protein synthesis functioning in the MAP kinase/ERK signaling pathway for cell division and differentiation, which also induces oncogenesis when mutated [12, 13]. The most common BRAF mutation is the V600E mutation with a 90% rate, which means glutamate is replaced by valine at codon 600 as a result of mismatch repair [13, 14]. As the aforementioned mutation takes place, uncontrolled activation of the RAS-RAF-MEK-ERK-MAP pathway results in CRCs with the common characteristics of right colon localization, microsatellite instability (MSI), mucinous type, poor prognosis, and aggressive tumors [12, 13, 15]. On the contrary, some recent studies report a bad prognosis for metastatic CRCs but no bad prognosis for early ones [16]. This finding does not affect the fact that new treatment modalities and emerging drugs are necessary for BRAF-mutated CRCs.

Ideas for new treatment options for BRAF-mutated CRCs concentrate on targeting the BRAF mutation because this mutation is blamed for the bad prognosis. Oncologists specializing in gastrointestinal tumors were lucky because the emerging therapy that they were seeking had already been invented to treat BRAF-mutated malignant melanoma under the name of “BRAF inhibitors.” Even the BRAF inhibitor treatment for CRCs is being debated currently and many clinical trials with preclinical studies are ongoing; this treatment is not approved by any authorized association. This case report presents our experience with a multidisciplinary treatment approach based on the BRAF inhibitor vemurafenib in a 59-year-old male with metastatic BRAF V600E mutant right colon adenocarcinoma.

## 2. Case Report

**Figure 1:**
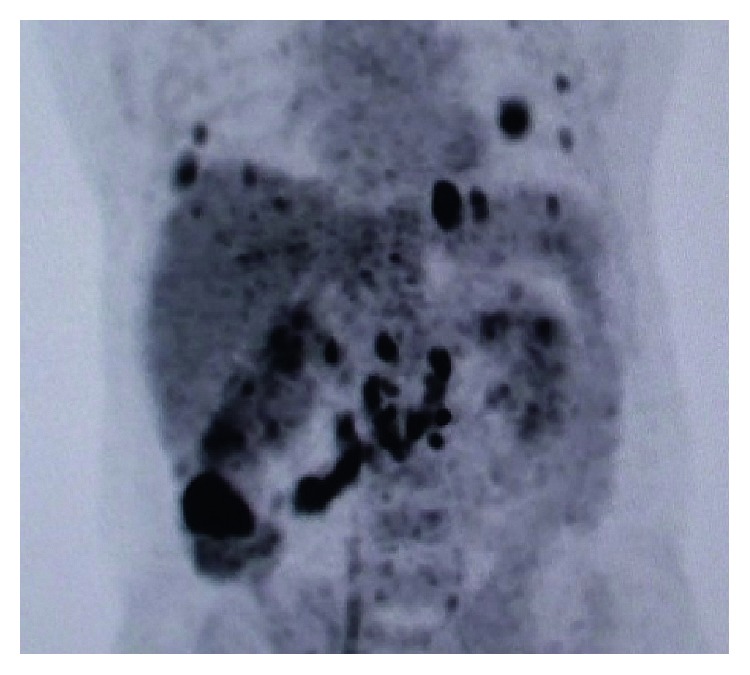
PET scan of the patient at the diagnosis of BRAF-mutant mCRC.

**Figure 2:**
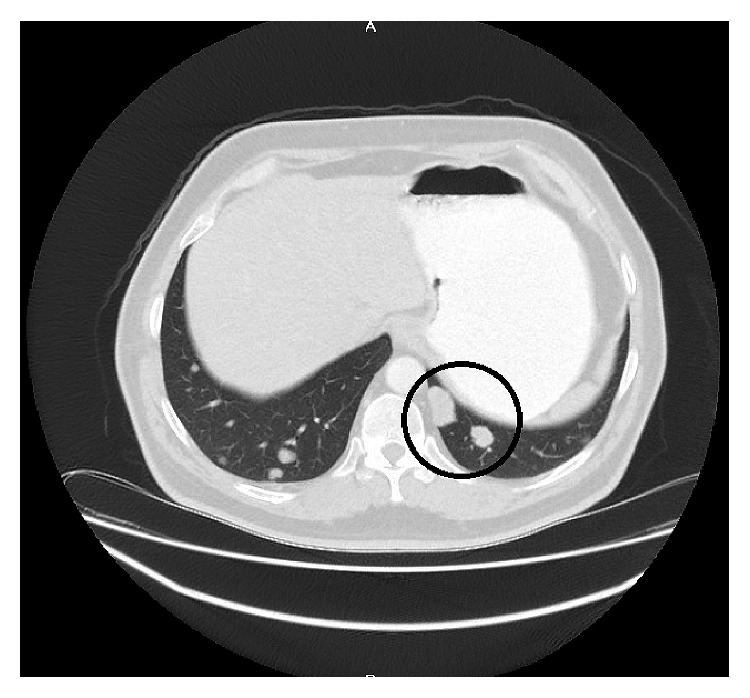
PET scan-correlated CT section of lung nodule at the diagnosis of BRAF-mutant mCRC.

A male patient with multiple coin lesions located in both lungs that were caught on incidental chest X-ray with a prediagnosis of metastatic cancer of unknown origin presented at our clinic. Thoracoabdominopelvic computed tomography (CT) with positron emission tomography (PET) scan and colonoscopic biopsy revealed a metastatic 5 × 4 cm right colon adenocarcinoma located 1 cm above the caecum with multiple sub-supracentimetric metastases located in both lungs and multiple metastatic mesenteric and interaortocaval/para-aortic/paracaval lymph nodes. Interestingly, there was no liver metastasis. The patient declared no symptoms until the incidental chest X-ray, which had been done only for check-up. He had only a history of weight loss of 5 kg in the last 2 months but was undergoing odontotherapy, causing him to eat less, which he declared as the probable reason for the weight loss. Tumor markers, complete blood count, and blood chemistry panel were normal at the time of diagnosis. A PET scan revealed bilateral multiple lung metastasis, the biggest being 2.5 cm with an SUVmax value of 12.61, metastasizing from the right colon mass (SUVmax value of 17.19) and with multiple positive lymph nodes (Figures 1 and 2).

The oncology council discussed the management of the patient's treatment. FOLFOX with bevacizumab was initiated as the first-line treatment, and the colonoscopic biopsy specimens were evaluated for histopathological patterns and gene mutations, because the tumor was diagnosed to be acting unusually. Advanced evaluation of the pathology specimens revealed wild-type *K-RAS*, wild-type *N*-*RAS*, V600E mutant BRAF, and microsatellite-stable adenocarcinoma of the colon in the same time, with the medication of the third dose of the FOLFOX + bevacizumab regimen.

The patient was resubmitted to the council for tailoring of the treatment according to the histopathological and genetic analysis of the specimen. The BRAF inhibitor vemurafenib at a classical dose of 960 mg twice per day peroral with a combination of infusional panitumumab 6 mg/kg biweekly was chosen for the patient according to the early results of currently ongoing clinical studies of vemurafenib in BRAF V600E mutated CRCs. Because the control CT after three doses of conventional chemotherapy showed a partial response to the tumor, the FOLFOX regimen without bevacizumab was also continued along with vemurafenib + panitumumab.

One week after the administration of the first dose of the FOLFOX + panitumumab + vemurafenib (FOLFOX-VEP), the patient's ALT-AST levels rose to over 100 U/L. Vemurafenib was interrupted for 5 days, and liver enzyme levels decreased to normal reference values, after which the drug was resumed. The FOLFOX in the FOLFOX-VEP regimen was deemed responsible for the increase in liver enzyme concentrations as a result of being combined with vemurafenib; because vemurafenib was believed to be more effective for tumor treatment than FOLFOX, the council decided to continue the chemotherapy as VEP without FOLFOX.

**Figure 3:**
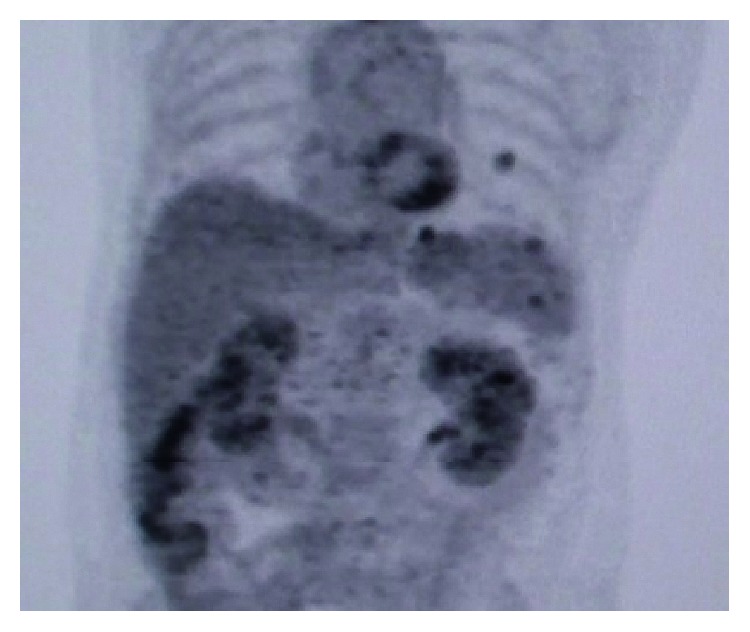
PET scan of the patient on day 45 of the vemurafenib treatment.

**Figure 4:**
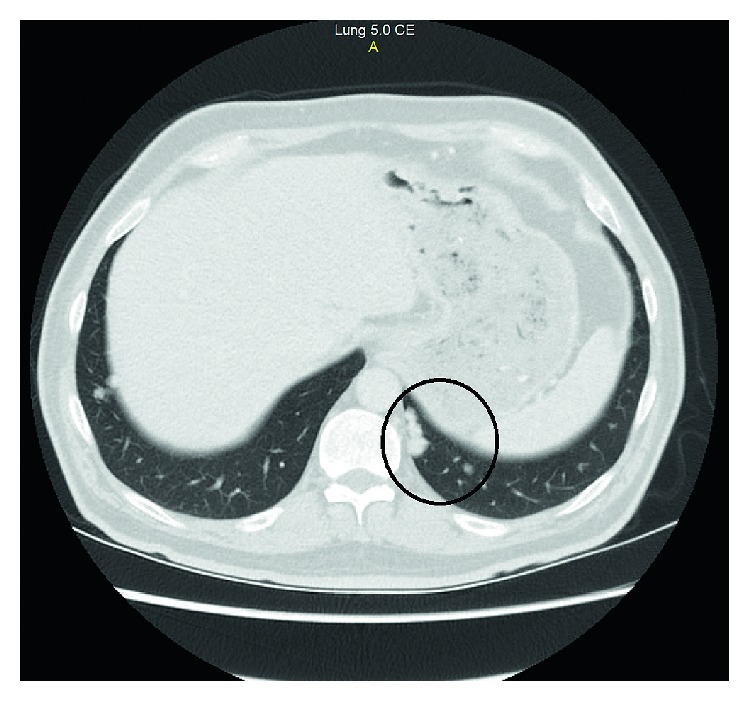
PET scan correlated CT section of lung nodule on day 45 of the vemurafenib treatment.

On day 45 of VEP medication, the patient had a PET scan for early evaluation of the novel treatment. The PET scan result was remarkable with a great response to the tumor (Figure 2). The SUVmax value of the primary tumor in the colon regressed to 5.5 from 17.19 with minimal or no uptake at other metastatic lesions. The regression of the tumor continued at the control PET scan which is performed 1 month after the previous one, and the PET scan resulted with 3.9 SUVmax value at the primary tumor site. (Figures 3 and 4).

The patient was planned for surgery, with the great response to the novel treatment. Four and a half months after diagnosis of the disease, a right hemicolectomy with mesocolic excision and interaortocaval/para-aortic/paracaval lymph node dissection was performed. Because the treatment applied to the patient had no cytotoxic effect, the targeted therapy was discontinued on preoperative day 4 and readministered on postoperative day 4. The patient was discharged on postoperative day 5 without any complications. Pathology reports were as follows: colonic mass of 2.5 × 1.5 cm with grade 2 regression due to neoadjuvant chemotherapy; 24 metastatic and 20 reactive lymph nodes; and stage 4, grade 2 mucinous adenocarcinoma (American Joint Committee on Cancer version 7 (2010)). An important point in the pathology report is the undifferentiated tumor reported at the initial pathology at the time of diagnosis was differentiated in the hemicolectomy specimen. Further histopathological and genetic evaluation was reported as follows: PIK3CA: negative; EGFR: negative; HER-2: negative; *K*-*RAS*: negative; *N*-*RAS*: negative; and BRAF V600E mutant adenocarcinoma.

The patient continued VEP treatment for another 45 days, and radiologic regression of the tumor continued; however, the biggest metastasis, measuring 1.7 cm and located in the lower right lung, showed signs of progression. The lesion was taken under radiologic control, and finally, right thoracotomy for multiple metastasectomy was planned and performed, yielding no lesions bigger than 0.5 cm. The pathology/histopathology/gene mutation report was the same as the abdominal pathology report.

Unfortunately, after the thoracal metastasectomy, the progression of the lesions continued. The remaining metastatic thoracal lesions that were progressing were shot with stereotactic body radiotherapy. The shot lesions regressed, but disease progressed, and the patient died of pneumonia 1 year after the thoracotomy. Survival of the patient was 19 months and 13 days.

## 3. Discussion

The BRAF mutation has a bad prognosis for advanced mCRCs, and those with microsatellite stable (MSS) have the worst prognosis, as in our case [16, 17]. Management of these metastatic patients is so difficult that many medical centers suggest no treatment because of the adverse effects of current chemotherapies and too little chance for cure or increased overall survival. Targeted therapy challenges this outlook, offering longer disease-free and overall survival and fewer adverse effects.

BRAF mutations are most commonly seen in malignant melanomas, and BRAF inhibitors, such as dabrafenib, sorafenib, vemurafenib, and encorafenib, are used in the treatment of malignant melanomas. As good results from the clinical trials of these drugs have been reported and BRAF inhibitors are approved for BRAF-mutant malignant melanoma by the Food and Drug Administration, new research for other BRAF-mutant cancers has begun, including CRCs, thyroid tumors, central nervous system tumors, and lung adenocarcinomas [18]. The CRC has progressed faster than the other tumors in terms of BRAF inhibitor treatment research. As the research for BRAF inhibitors in BRAF-mutant CRCs continues, results from clinical experiences and researches with BRAF inhibitors used to treat BRAF-mutant malignant melanoma showed that drug resistance may also occur at the treatment of BRAF mutant CRC as it occurred at BRAF mutant malign melanoma [19]. Researchers have suggested for the use of MEK inhibitors to overcome the resistance mechanism [20, 21]. This has mostly worked in malignant melanoma, but no large series are available yet. Results for the BRAF inhibitor + MEK inhibitor combination for BRAF-mutant mCRCs are not well known, but data in case reports seem promising [22]. The combination is so promising that a phase 1-2 clinical trial for the combination of BRAF/MEK/EGFR inhibitors with 5-FU for treatment of BRAF-mutant mCRCs is still ongoing; the combination of these four drugs seems the most sensible one.

Drug resistance is the biggest problem for oncologists, as mentioned earlier. Although vemurafenib had a great response in BRAF-mutant mCRCs, drug resistance may cause mortal results as in our case. Alternatives to targeted therapies with BRAF inhibitors are programmed cell death ligand inhibitors or named checkpoint inhibitors. These drugs are also part of the targeted therapy, but unlike BRAF inhibitors, they are not specific to tumor mutations and inhibit the programmed cell death receptors of the tumor cells, providing T lymphocytes to kill them. Unfortunately, drug resistance has also occurred with unknown complex mechanisms for checkpoint inhibitors, but they are still a glimmer of hope [23]. Sehdev et al. found a pathologically complete response in a BRAF-mutated, high-MSI patient using checkpoint inhibitor therapy and Corcoran et al. reported a complete response in one patient using a BRAF + MEK inhibitor, but resistance is still a problem for all types of targeted therapies [24, 25].

Targeted therapies may provide better tumor response and prolonged overall survival, but their main advantage is that they provide these benefits with fewer adverse effects and increased quality of life for the patients undergoing chemotherapy. The most common adverse effects of BRAF inhibitors are dermatologic [26]. Gastrointestinal, ocular, hematological, and constitutional side effects are rare. Our patient had only grade 1 side effects: fatigue and liver enzyme elevation. During the BRAF inhibitor administration, the patient was kept away from the sun to prevent cutaneous adverse effects. As a result of minimalized adverse effects, our patient, a surgeon himself, continued his routine social and academic life during the targeted therapy, even after the surgery, which is the most important advantage of targeted therapy.

During the treatment, our patient had a major abdominal surgery followed immediately by thoracotomy. While preparing for surgery, the patient was given EGFR and BRAF inhibitors. The medication was interrupted 4 days before surgery and readministered 4 days after surgery because no hematologically known adverse effect was present, and BRAF and EGFR inhibitors have no known adverse effect on wound healing. No complications or drug interaction occurred during or after the surgery. This indicates that the BRAF inhibitor vemurafenib in combination with the EGFR inhibitor panitumumab may be continued during surgery if the patient is amenable to surgery.

Abdominal surgery and multisite lung metastasectomy have been contraindications for mCRCs for many years; however, this is now being debated. Intrahepatic metastasectomy may be more acceptable, but extrahepatic metastasectomy remains controversial. Wei et al. state that extrahepatic metastasectomy for mCRCs is safe, but relapse commonly occurs within a short time; however, they suggest aggressive extrahepatic metastasectomy in selected patients for increased survival [27]. In addition to Wei et al., Vodicka et al. and many other researchers suggest pulmonary metastasectomy for advanced cancers [28, 29]. In our case, we aimed at a cure for our patient before the probable drug resistance to vemurafenib could occur. For this reason, we planned and applied consecutive abdominal and thoracal surgeries, but the drug resistance occurred just at the time of vemurafenib drug resistance began which was around the 6th month of the vemurafenib treatment but not later [30]. To reduce the risk of drug resistance or at least to delay it, MEK inhibitors are suggested to be combined with BRAF inhibitors for malignant melanoma. Corcoran et al. had no drug resistance in a patient with BRAF-mutant mCRCs when using the combination of BRAF and MEK inhibitors [24]. We also think that this combination may be beneficial for patients and await the results of the phase studies that are being conducted.

Herr et al. state that BRAF inhibitors induce epithelial differentiation, as in our case [31]. Undifferentiated tumor found in the colonoscopic biopsy had transformed to differentiated tumor in the hemicolectomy specimen, which decreased the Ki-67 score of the tumor to 50% from 85%. This parameter also shows how BRAF inhibitors are effective in BRAF-mutant CRCs.

To our knowledge and from the literature survey, it seems that BRAF-mutant mCRCs constitute the most difficult CRCs to treat, but emerging targeted therapies tailored according to tumor mutation profile may take the place of oncologic surgery due to their incredible results, including complete responses and tolerable adverse effects. The drugs that will probably provide this will be mutation-specific inhibitors, such as BRAF and MEK inhibitors, and programmed cell death ligand inhibitors.

In our case, we did not have complete response but did have overall survival of more than 19 months, which is significantly higher than expected. Sufficient data were not available in the literature to date on the treatment of patients for BRAF inhibitor resistance, but better results with BRAF inhibitors + MEK inhibitors than with BRAF inhibitors alone in both BRAF-mutant mCRCs and malignant melanoma show that BRAF inhibitors with reduced drug resistance through the help of MEK inhibitors offer a feasible treatment for BRAF-mutant cancers such as mCRCs.
